# Descriptive analysis of cochrane child-relevant systematic reviews: an update and comparison between 2009 and 2013

**DOI:** 10.1186/s12887-017-0908-7

**Published:** 2017-07-11

**Authors:** Katelynn Crick, Denise Thomson, Ricardo M. Fernandes, Megan Nuspl, Dean T. Eurich, Brian H. Rowe, Lisa Hartling

**Affiliations:** 1 School of Public Health, University of Alberta, Edmonton, Canada; 2 Cochrane Child Health, The Cochrane Collaboration, Department of Pediatrics, University of Alberta, Edmonton, Canada; 3 Department of Pediatrics, Hospital Santa Maria (CHLN), Lisbon Academic Medical Center, Lisbon, Portugal; 4 Alberta Research Centre for Health Evidence, Department of Pediatrics, University of Alberta, Edmonton, Canada; 5 Clinical Pharmacology Unit, Instituto de Medicina Molecular, University of Lisbon, Lisbon, Portugal; 6 Department of Emergency Medicine, University of Alberta, Edmonton, Canada; 7 Emergency Strategic Clinical Network, Alberta Health Services, Edmonton, AB Canada; 8 4-498B Edmonton Clinic Health Academy, 11405 – 87 Avenue, Edmonton, AB T6G 1C9 Canada

**Keywords:** Systematic reviews, Meta-analyses, Child, Pediatrics, Reporting quality, Methods

## Abstract

**Background:**

Systematic reviews support health systems and clinical decision-making by identifying and summarizing all existing studies on a particular topic. In 2009, a comprehensive description of child-relevant systematic reviews published in the Cochrane Database of Systematic Reviews was compiled. This study aims to provide an update, and to describe these systematic reviews according to their content and methodological approaches.

**Methods:**

All child-relevant systematic reviews published by the Cochrane Collaboration in the Cochrane Database of Systematic Reviews (CDSR) as of March, 2013 were identified and described in relation to their content and methodological approaches. This step equated to an update of the Child Health Field Review Register (CHFRR). The content of the updated CHFRR was compared to the published 2009 CHFRR description regarding clinical and methodological characteristics, using bivariate analyses. As the Cochrane Collaboration has recognized that disease burden should guide research prioritization, we extracted data from the Global and National Burden of Diseases and Injuries Among Children and Adolescents Between 1990 and 2013 study in order to map the distribution of the burden of disease in child health to the distribution of evidence across Review Groups in the CHFRR.

**Results:**

Of the 5,520 potential Cochrane systematic reviews identified, 1,293 (23.4%) were child-relevant (an increase of 24% since 2009). Overall, these reviews included 16,738 primary studies. The most commonly represented Review Groups were Airways (11.5%), Cystic Fibrosis and Genetic Diseases (7.9%), Acute Respiratory Infections (7.8%), Developmental, Psychological and Learning Problems (6.7%), and Infectious Diseases (6.2%). Corresponding authors were most often from Europe (51%), North America (15%), and Australia (15%). The majority of systematic reviews examined pharmacological interventions alone (52% compared to 59% in 2009). Out of 611 reviews that were assessed as up-to-date, GRADE was used in 204 (35%) reviews to assess the overall quality of the evidence, which was often moderate (35.6%) or low (37.8%) for primary outcomes. Ninety percent of reviews that were assessed as up to date used the Cochrane Risk of Bias tool, or a modified version, to assess methodological quality. Most reviews conducted one or more meta-analyses (73%). Among the 25 leading causes of death globally, the Review Groups associated with the largest number of causes were: 1) Infectious Diseases, 2) Anaesthesia, Critical, and Emergency Care, 3) Injuries, 4) Pregnancy and Childbirth (PC), and 5) Neonatal. There were large discrepancies between the number of causes of mortality that each Review Group was associated with and the total amount of evidence each Review Group contributed to the CHFRR. Ninety-eight percent of the causes of mortality in 2013 were from developing nations, but only 224 (17.3%) reviews had corresponding authors from developing countries.

**Conclusion:**

The content and methodological characteristics of child-relevant systematic reviews in the Cochrane CHFRR have been described in detail. There were modest advances in methods between 2009 and 2013. Systematic reviews contained in the CDSR offer an important resource for researcher’s, clinicians and policy makers by synthesizing an extensive body of primary research. Further content analysis will allow the identification of clinical topics of greatest priority for future systematic reviews in child health.

**Electronic supplementary material:**

The online version of this article (doi:10.1186/s12887-017-0908-7) contains supplementary material, which is available to authorized users.

## Background

Systematic reviews (SRs) facilitate decision-making by considering all evidence on a specific question of interest (e.g., intervention effectiveness, diagnostic test accuracy). Systematic reviews, “identify, appraise, and synthesize research-based evidence,” and present it in an accessible format for potential use by healthcare providers, consumers, researchers, and policy makers [1]. Cochrane SRs are published online in the Cochrane Database of Systematic Reviews (CDSR), a central component of the Cochrane Library. The Cochrane Collaboration is structured around 52 Cochrane Review Groups (CRGs) which are responsible for producing and maintaining Cochrane reviews within each of their particular areas of health.

A comprehensive identification and depiction of child-relevant systematic reviews in the CDSR was compiled in 2009, called the Child Health Field Review Register (CHFRR) [2]. At that time, the description by Bow et al. identified the scope of child-relevant research evidence accessible in Cochrane reviews and important gaps in research. Additionally, the description of methodological approaches used in SRs contained in the CHFRR provided knowledge on the rigor and consistency of the identified reviews [2].

Following the methods of Bow et al., this study aims to provide a more recent analysis of the CHFRR by identifying all child-relevant SRs in the CDSR as of March, 2013 and to depict these SRs in terms of their content and methodological approaches [2]. A more recent analysis of the CHFRR and a comparison of it’s results to the findings of 2009 will help to identify: 1) the scope of child-relevant evidence accessible in SRs; 2) gaps in research evidence and changes since 2009; 3) methodological advancements; and 4) limitations and inconsistencies in SRs.

## Methods

### Definition of child-relevant SRs

Following the methodology of Bow et al. and the criteria of the Cochrane Child Health Field, we defined child-relevant SRs as those that intended to include children (regardless of whether adults were also included) or reviewed an intervention that was not applied directly to children, but was intended to improve the health and well-being of children (e.g., smoking cessation programs for families [3], psychological education for siblings of children with severe mental illness [4], interventions to improve in-hospital antibiotic prescribing practices [5]). Bow [2] SRs related to neonates were not included; these reviews are captured by a single Review Group (Cochrane Neonatal) while the intent of the CHFRR was to identify and describe the child-relevant reviews that are prepared by and scattered across numerous other Review Groups. We excluded SRs relevant to pregnancy except for studies on breastfeeding or nutritional supplements during pregnancy as these reviews contain outcomes that are relevant to child health [2].

### Identification of child-relevant SRs

Child-relevant SRs as of March, 2013 were identified using the existing tagging system of the CHFRR. The search methodology (Additional file 1) and screening algorithm (Additional file 2) used to tag SRs as child-relevant in the CDSR is the same as that which was used by Bow et al., which involves searching the CDSR using a pediatric search filter and a pre-determined screening algorithm [2].

### Data extraction

An electronic REDCap [6] (Vanderbilt University, Nashville, Tennessee, USA) form was developed and pilot tested for data extraction (available by request from corresponding author). Data were extracted from the identified SRs and entered onto the REDCap form. Following the methodology of Bow et al., the variables extracted fell into three main categories: general review and author characteristics, characteristics of included studies, and methodological approaches. General review characteristics included publication dates, country of primary author, nature of interventions (pharmacological vs. non-pharmacological), and external sources of funding [2]. The country of corresponding authors was classified on income level (high, upper-middle, lower-middle, or low income) [7]. The nature of interventions (pharmacological vs. non-pharmacological) under comparison was classified using the Health Canada definition [8]. Standard Health Canada definitions were also used to categorize interventions as a natural health product or a device [9, 10]. Analysis of the characteristics of included studies involved examining the primary study designs sought and included in each review, the number of participants, and the ages represented [2]. Primary studies were categorized as including child participants only (all participants <18 years of age), adults only (all participants ≥18 years of age), or mixed [2]. Methodological approaches included whether reviews indicated a primary outcome, authors methodological approach to quality assessment, analytic approach, whether author’s conducted meta-analyses, the proportion of primary studies included in the largest meta-analysis, and whether publication bias was evaluated [2].

Baseline data from the 2009 study were extracted and recorded directly onto a Microsoft Excel**®** (Version 14.7.0) form for comparison with the 2013 data. Variables extracted were identical to those extracted from the reviews for the current study, where applicable.

Based on feedback during peer-review, we added the following consideration and relevant analysis to examine the content of the CHFRR relative to the global burden of disease. The Cochrane Collaboration has recognized that disease burden should guide research prioritization, with more disabling diseases having a greater representation in the CDSR [11]. Mortality is frequently used as an index of burden of disease and is, “used to assess and compare the relative impact of different diseases and injuries on population health” [12]. In order to map the distribution of the burden of disease in child health to the distribution of evidence across Review Groups in the CHFRR, we extracted data from the Global and National Burden of Diseases and Injuries Among Children and Adolescents Between 1990 and 2013 study [13].

### Data analysis

Univariate analyses were carried out to describe the reviews contained in this sample and the primary studies they included. We analysed the data overall (across all Review Groups) and within subgroups based on the relevant Review Groups, which cover different clinical areas (listing of Cochrane Review Groups can be found at http://www.cochranelibrary.com/about/cochrane-review-groups.html) [14]. We presented data separately for the 5 Review Groups which had produced the largest number of child-relevant reviews. Dichotomous variables were described as counts and percentages. Continuous variables were described as means and standard deviations (SDs) in the case of normally distributed data, or medians and interquartile ranges (IQRs) in the case of skewed data.

Bivariate analyses (chi-squared test, two proportion z-test, and Wilcoxon-Mann-Whitney test, where appropriate) were used to compare the 2009 and 2013 results. Data were compared between the two groups (2009 and 2013) within each of the 3 main categories of variables extracted: general review characteristics, characteristics of included studies, and methodological approaches. In order to control for multiplicity, an issue that arises when looking for similarities and differences between the same groups on multiple measures, the Bonferroni method was used to calculate a corrected significance level [15, 16]. We considered *P*-values less than 0.001 significant for these comparisons.

The proportion of mortality attributable to the top 25 leading causes of death globally, in developing countries, and developed countries were compared descriptively to the proportion of evidence (i.e., number of systematic reviews) in the CHFRR. We assigned Review Groups that could be applicable to each of the top 25 leading causes of global mortality. For each leading cause, we then summed the total number of child-relevant reviews contained in each of the applicable Review Groups. Additionally, we calculated the total proportion of potentially applicable evidence across the identified Review Groups for each of the 25 leading causes of mortality. We also ranked the leading causes of death (among the top 25 globally) for developing and developed nations. We calculated the total number of causes each of the identified Review Groups was associated with, out of the top 25 leading causes, and calculated the proportion of causes each of the identified Review Groups was applicable to. We then compared the proportion of causes each of the identified Review Groups was associated with to the proportion of evidence that each of the identified Review Groups contributed to the CHFRR.

## Results

**Fig. 1:**
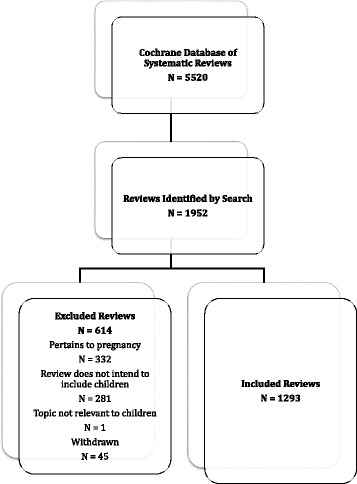
Flow of systematic reviews through the screening process

Of the 5520 reviews listed in the CDSR as of March 2013, 1338 (24.2%) were identified as child-relevant and were published by 45 separate Cochrane Review Groups (Fig. 1). Forty-five of the identified reviews were labelled as withdrawn, however, and therefore excluded from the analysis (*n* = 1293; 23.4%). The 5 Review Groups producing the largest amount of child-relevant reviews were: Airways (*n* = 149 representing 11.5% of all included child-relevant reviews), Cystic Fibrosis and Genetic Diseases (CF and Genetic Diseases) (*n* = 103; 8.0%); Acute Respiratory Infections (ARI) (*n* = 100; 7.7%), Developmental, Psychological and Learning Problems (DPLP) (*n* = 86; 6.7%), and Infectious Diseases (ID) (*n* = 79; 6.1%). Further details of child-relevant reviews, including the percentage of reviews within each Review Group in 2009 and 2013, can be found in Table 1.

**Table 1:** Child-relevant reviews by the 10 largest Cochrane Collaborative Review Groups (CRGs)

Cochrane Collaboration Review Group	Total completed reviews in CDSR		Total child-relevant reviews (percent of child relevant to all completed reviews)		Percent change in total child-relevant reviews between 2009 and 2013
	2009	2013	2009	2013	%
Acute Respiratory Infections	102	141	70 (68.6)	115 (81.6)	64.3
Airways	221	266	118 (53.4)	151 (56.8)	28
Cystic Fibrosis and Genetic Diseases	80	119	66 (82.5)	103 (86.6)	56.1
Developmental, Psychological, and Learning Problems	73	115	49 (67.1)	87 (75.7)	77.6
Ear, Nose and Throat Disorders	55	89	28 (50.9)	48 (53.9)	71.4
Epilepsy	50	71	30 (60.0)	51 (71.8)	70
HIV/AIDS	57	98	22 (38.6)	43 (43.9)	95.5
Infectious Diseases	94	116	58 (61.7)	83 (71.6)	43.1
Oral Health	92	135	32 (33.7)	49 (36.3)	53.1
Pregnancy and Childbirth	359	517	26 (7.2)	51 (9.9)	96.2

^a^Table excludes reviews related to neonates and pregnancy, except for reviews on breastfeeding or nutritional supplements during pregnancy

### General characteristics of child-relevant reviews

The median year of protocol publication and full review publication in the CHFRR was 2004 and 2007, respectively. The median amount of time between protocol and review publication in years was 2 (IQR: 1, 3). The median year in which reviews were ‘last assessed as up-to-date’ was 2010 (IQR: 2008, 2011). Seven-hundred and twenty-one (55.8%) reviews were last assessed as up to date prior to 2010 (Table 2).

**Table 2:** General characteristics of child-relevant reviews, overall and for the five CRGs containing the largest number of child-relevant reviews

	Overall *n* = 1293	Airways *n* = 148	Cystic Fibrosis and Genetic Diseases *n* = 102	Acute Respiratory Infections *n* = 100	Developmental, Psychological, and Learning Problems *n* = 86	Infectious Diseases *n* = 79
Publication Characteristics						
Year protocol published (median)	2004	2001	2003	2004	2006	2003
Year review published (median)	2007	2003	2006	2007	2008	2006
Number of years between publication of protocol and review (median [IQR])	2 (0,4)	2 (1,2)	1 (1,2)	2 (2,3)	1.5 (1,2)	2 (1,3)
Year last assessed as up-to-date (median)	2010	2009	2011	2011	2010	2009
Country Classification of corresponding author: income level (World Bank: http://www.worldbank.org/), n (% total)						
High income	1066 (83.5)	140 (95.2)	87 (87.9)	68 (68.7)	74 (86.1)	45 (59.2)
Upper middle income	142 (11.1)	5 (3.4)	7 (7.1)	20 (20.2)	9 (10.5)	10 (13.2)
Lower middle income	62 (4.9)	2 (1.4)	5 (5.1)	11 (11.1)	3 (3.5)	18 (23.7)
Low income	6 (0.5)	0 (0.0)	0 (0.0)	0 (0.0)	0 (0.0)	3 (4.0)
Nature of intervention: classification 1, n (% total)						
Pharmacological	666 (51.8)	107 (72.3)	73 (74.5)	61 (61.0)	27 (31.4)	39 (49.4)
Non-pharmacological	466 (36.2)	37 (25.0)	22 (22.5)	24 (24.0)	57 (66.3)	28 (35.4)
Both pharmacological and non-pharmacological	155 (12.0)	4 (2.7)	3 (3.1)	15 (15.0)	2 (2.3)	12 (15.2)
Nature of intervention: classification 2, n (% total)						
Drug	575 (44.5)	98 (66.2)	58 (56.9)	49 (49.0)	14 (16.3)	38 (48.1)
Surgical	53 (4.1)	2 (1.4)	5 (4.9)	0 (0.0)	2 (2.3)	2 (2.5)
Behavioural/educational/psychological	169 (13.1)	15 (10.1)	7 (6.9)	4 (4.0)	43 (50.0)	3 (3.8)
Device	96 (7.4)	13 (8.8)	5 (4.9)	2 (2.0)	4 (4.7)	2 (2.5)
Natural health product	120 (9.3)	7 (4.7)	3 (2.9)	22 (22.0)	15 (17.4)	5 (6.3)
Vaccine	37 (2.9)	4 (2.7)	4 (3.9)	8 (8.0)	0 (0.0)	12 (15.2)
Non-surgical clinical practice or procedure	207 (16.0)	6 (4.1)	20 (19.6)	13 (13.0)	7 (8.1)	14 (17.7)
Policy or legislation	36 (2.8)	3 (2.0)	0 (0.0)	2 (2.0)	1 (1.2)	3 (3.8)
External source of funding, n (% total)						
Not stated	262 (20.3)	18 (12.2)	57 (55.9)	13 (13.0)	13 (15.1)	5 (6.3)
No	331 (25.6)	26 (17.6)	17 (16.7)	34 (34.0)	15 (17.4)	4 (5.1)
Yes	700 (54.1)	104 (70.3)	28 (27.5)	53 (53.0)	58 (67.4)	70 (88.6)
Cochrane	142 (11.0)	19 (12.8)	3 (2.9)	12 (12.0)	10 (11.6)	7 (8.9)
Academic	83 (6.4)	1 (0.7)	3 (2.9)	11 (11.0)	4 (4.7)	6 (7.6)
Government	461 (35.7)	79 (53.4)	14 (13.7)	26 (26.0)	33 (38.4)	62 (78.5)
Industry	7 (0.5)	0 (0.0)	0 (0.0)	0 (0.0)	1 (1.2)	1 (1.3)
Non-profit organization	235 (18.2)	43 (29.1)	15 (14.7)	12 (12.0)	24 (27.9)	10 (12.7)
Other	21 (1.6)	2 (1.4)	0 (0.0)	2 (2.0)	1 (1.2)	0 (0.0)

Most frequently, primary authors were from the following continents: Europe (653; 48.8%) (UK [368; 28.5%] followed by the rest of Europe [285; 22.0%]), North America (193; 14.9%) (Canada [99; 7.7%], USA [86; 6.7%]), Australia (190; 14.7%), Asia (150; 11.6%), Africa (61; 4.7%), and South America (46; 3.6%). A large majority of reviews had corresponding authors from high-income countries (1069; 82.7%).

Over half of reviews examined pharmacological interventions (669; 51.7%) based on the Health Canada definition. Among reviews examining pharmacological interventions, the most commonly studied types of interventions included drugs (617; 92.2%), natural health products (55; 8.2%), and vaccines (13; 1.9%). The majority of the remaining reviews examined non-pharmacological interventions (468; 36.2%). A small proportion of reviews examined both pharmacological and non-pharmacological interventions (156; 12.1%). Seventeen (1.3%) diagnostic reviews were identified.

The majority of reviews had one or more external sources of funding (700; 54.1%). The median number of external sources of funding per review was 1 (IQR: 0, 2). Of the reviews having one or more external sources of funding, the majority were funded by government sources (461; 35.7%), followed by non-profit organizations and foundations (235; 18.2%), the Cochrane Collaboration (142; 11.0%), and academic sources (83; 6.4%).

### Characteristics of studies included in child-relevant reviews

Most commonly, reviews planned to include randomized controlled trials (RCTs) and no other study designs (698; 54.0%). A large proportion of reviews intended to include RCTs and other designs (503; 38.9%) (observational and quasi-experimental designs), while very few reviews intended to include solely other designs (4; 0.3%). Of the reviews intending to include solely other designs, half (2; 50%) were reviews of patient-level interventions, 1 (25%) was a community-based intervention, and 1 (25%) was a review of diagnostic test accuracy (Table 3).

**Table 3:** Characteristics of studies included in child-relevant reviews, overall and for the 5 CRGs containing the largest number of child-relevant reviews

	Overall *n* = 1293	Airways *n* = 148	Cystic Fibrosis and Genetic Diseases *n* = 102	Acute Respiratory Infections *n* = 100	Developmental, Psychological, and Learning Problems *n* = 86	Infectious Diseases *n* = 79
Study Designs						
RCTs only (intended), n (% total)	695 (57.7)	123 (85.4)	22 (78.6)	66 (66.7)	33 (39.8)	44 (55.7)
RCTs only (actual), n (% of included studies)	844 (73.5)	123 (93.9)	72 (98.6)	62 (66.7)	46 (66.7)	53 (68.8)
RCTs and other designs (intended), n (% total)	502 (41.7)	19 (13.2)	5 (17.9)	33 (33.3)	50 (60.2)	33 (41.8)
RCTs and other designs (actual), n (% of included studies)	270 (23.5)	7 (5.3)	1 (1.4)	29 (31.2)	23 (33.3)	20 (26.0)
Non-RCTs (intended), n (% total)	8 (0.7)	2 (1.4)	1 (3.6)	0 (0.0)	0 (0.0)	2 (2.5)
Non-RCTs (actual), n (% of included studies)	34 (3.0)	1 (0.8)	0 (0.0)	2 (2.2)	0 (0.0)	4 (5.2)
Studies and Participants						
Number of studies included (median, IQR)	8 (4, 17)	8.5 (4, 21)	4 (2, 8)	8.5 (5, 18)	8 (4, 11)	10.5 (7, 21)
Reviews with no relevant studies, n (% of reviews in group)	127 (9.8)	17 (11.5)	27 (26.5)	4 (4.0)	15 (17.9)	1 (1.3)
Child only studies [% of total number of studies]	21.2	26.3	24.5	31.9	19.6	34.6
Adult only studies [% of total number of studies]	25.2	26.3	9.1	15	3.4	17.7
Mixed child and adult studies [% of total number of studies]	17.1	24.3	42.4	8.3	9.1	30.7
Number of participants included (median, IQR)	980 (278, 3394)	767 (234, 3747)	213 (107, 397)	1906 (618, 4414)	746 (201, 1509)	2730 (1283, 9128)

Regarding study identification and inclusion, 127 (9.8%) literature searches returned no relevant studies meeting the review inclusion criteria (so-called ‘empty reviews’). The proportion of empty reviews (reviews with no applicable studies) was particularly high in the Cystic Fibrosis and Genetic Disorders (27; 21.3%) Review Group, Airways (17; 13.4%), and the Developmental, Psychosocial, and Learning Problems (15; 11.8%) Review Group. Of the 1164 (90.0%) reviews including at least one study, 851 (73.1%) included RCTs only, 272 (23.4%) included both RCTs and other study designs, and 34 (2.9%) included solely other designs.

The median number of studies included per review was 8 (IQR 4, 17). Child-only reviews made up 283 (21.9%) of the included reviews. Seven (0.5%) of the reviews included adult-participants only, and 1003 (77.6%) of the reviews included both child and adult participants. A median of 980 (IQR: 278, 3394) participants were included per review.

### Methodological approaches in child-relevant reviews

Reviewers identified a primary outcome in 1040 (82.4%) of reviews. Among the 524 reviews that were assessed as up-to-date, and which contained at least one RCT, the Cochrane Risk of Bias (RoB) tool was the most commonly used approach to assess methodological quality (454 (86.6%) used the RoB tool and 39 (7.4%) used a modified version of the RoB tool). The modified versions of the RoB tool were most frequently modified in attempt to assess observational or quasi-experimental designs. Seventy-four (10.5%) reviews used both the RoB tool and at least one other approach to assessing methodological quality. The Jadad scale was used in 99 (7.7%) reviews and allocation concealment alone (independent of the RoB tool) was used in 329 (25.4%) reviews. Among reviews assessed as up-to-date, 65 (11.5%) reviews used more than one approach to assessing methodological quality (Table 4).

**Table 4:** Methodological approaches in child-relevant reviews, overall and for the 5 CRGs containing the largest number of child-relevant reviews

	Overall *n* = 1293	Airways *n* = 148	Cystic Fibrosis and Genetic Diseases *n* = 102	Acute Respiratory Infections *n* = 100	Developmental, Psychological, and Learning Problems *n* = 86	Infectious Diseases *n* = 79
Outcomes						
Reviewers specified one or more primary outcomes, n (%)	1040 (82.3)	116 (78.9)	91 (100.0)	91 (91.0)	60 (71.4)	70 (88.6)
Assessment of methodological quality in SR’s with included studies						
Risk of Bias, n (%)	687 (56.4)	65 (44.8)	55 (73.3)	85 (87.6)	47 (66.2)	36 (45.6)
Modified Risk of Bias, n (%)	94 (7.7)	5 (3.5)	15 (20.0)	1 (1.0)	2 (2.8)	0 (0.0)
Jadad, n (%)	99 (7.4)	62 (41.9)	2 (2.0)	2 (2.0)	1 (1.2)	1 (1.3)
Allocation concealment, n (%)	329 (24.6)	67 (45.3)	7 (6.9)	7 (7.0)	21 (24.4)	2 (2.5)
Other tool, n (%)	209 (15.6)	13 (8.8)	2 (2.0)	6 (6.0)	13 (15.1)	43 (54.4)
Analysis						
Children analyzed separately in reviews with both child and adult participants, n (%)						
Yes	88 (10.0)	17 (17.0)	0 (0.0)	10 (18.5)	2 (6.7)	4 (6.2)
No	688 (78.5)	73 (73.0)	65 (97.0)	42 (77.8)	26 (86.7)	58 (89.2)
N/A	101 (11.5)	10 (10.0)	2 (3.0)	2 (3.7)	2 (6.7)	3 (4.6)
Subgroup analyses for children in reviews with both child and adult participants, n (%)						
Yes	121 (13.9)	42 (44.2)	5 (7.5)	7 (13.0)	5 (16.7)	9 (13.9)
No	647 (74.4)	42 (44.2)	60 (89.6)	45 (83.3)	23 (76.7)	53 (81.5)
N/A	102 (11.7)	11 (11.6)	2 (3.0)	2 (3.7)	2 (6.7)	3 (4.6)
Subgroup analyses within children in any review with children included, n (%)						
Yes	284 (23.3)	27 (18.4)	9 (12.0)	39 (40.2)	17 (24.3)	45 (57.0)
No	824 (67.7)	106 (72.1)	65 (86.7)	56 (57.7)	51 (72.9)	31 (39.2)
N/A	110 (9.0)	14 (9.5)	1 (1.3)	2 (2.1)	2 (2.9)	3 (3.8)
Publication bias assessed, n (%)						
Yes	149 (13.8)	20 (17.2)	0 (0.0)	19 (20.7)	9 (14.1)	13 (16.9)
No	492 (45.7)	50 (43.1)	27 (42.2)	28 (30.4)	21 (32.8)	50 (64.9)
Discussed, but not formally assessed	114 (10.6)	16 (13.8)	8 (12.5)	9 (9.8)	4 (6.3)	4 (5.2)
Planned, but not assessed	322 (29.9)	30 (25.9)	29 (45.3)	36 (39.1)	30 (46.9)	10 (13.0)
Meta-analysis conducted in those reviews with included studies, n (%)	784 (72.7)	98 (84.5)	38 (59.4)	75 (81.5)	47 (72.3)	65 (84.4)
Number of studies contributing to meta-analysis (median, IQR)	5 (3, 10)	6 (4, 15)	2 (2, 3)	5 (3, 8)	5 (3, 7)	6 (3, 9)
Percentage of included studies contributing to meta-analysis (in reviews that conducted a meta-analysis) (median, IQR)	45.5 (28.6, 70.0)	50.0 (31.6, 66.7)	44.4 (25.0, 66.7)	50.0 (33.3, 75.0)	61.0 (37.5, 66.7)	37.5 (28.6, 60.0)

Among 1003 reviews that included mixed participants (children and adults), 51 (5.1%) planned to analyze children separately from adults and 202 (20.1%) planned to analyze children using subgroups. In actuality, 60 (6.0%) reviews analyzed children separately from adults and 99 (9.9%) analyzed children and adults using subgroups. Subgroup analyses were performed within 283 (22.0%) reviews. Among reviews reporting subgroup analyses within children, 71 (5.5%) reviews based the subgroups on age.

Statistical tests for funnel plot asymmetry for small-study effects are recommended by the Cochrane Collaboration for use as long as there are 10 or more studies included in a meta-analysis [17]. Out of 111 reviews that contained a meta-analysis with 10 or more studies, publication bias was formally assessed in 57 (51.4%) reviews. Sixteen (14.4%) of the eligible reviews planned to formally assess publication bias but did not carry out the analysis; ten (9%) reviews discussed publication bias, but did not formally assess it, and one quarter of reviews (25.2%) did not discuss publication bias anywhere in the review. Among reviews formally assessing publication bias, 11 (19.3%) reviews assessed it graphically and statistically, while 45 (78.9%) reviews assessed it solely using graphical methods, and 1 (1.7%) review assessed it solely using statistical methods.

Among the 611 reviews that were assessed as up-to-date, the Grading of Recommendations Assessment, Development and Evaluation (GRADE) approach was used in 204 (35.4%) reviews to assess the quality of the evidence of the literature included in the review. Among the up-to-date reviews using GRADE, 181 (88.7%) provided a GRADE assessment for the primary outcome of the review. Of primary outcomes that were given a GRADE, 68 (37.8%) were assessed a GRADE of low, 64 (35.6%) a GRADE of moderate, 26 (14.4%) a GRADE of high, and 22 (12.2%) a GRADE of very low.

Meta-analyses were performed in 784 (72.7%) reviews. Amongst reviews that executed one or more meta-analyses, a median of 5 (IQR: 3, 10) trials comprised the largest meta-analysis conducted in each review.

### 2009 vs. 2013 comparison

The total number of reviews included in the CDSR increased by 41% from 2009 (*n* = 3916) to 2013 (*n* = 5520), while the number of child-relevant reviews in the CHFRR increased by 24% (2009: *n* = 1046; 2013: *n* = 1293). The 5 CRGs producing the largest number of child-relevant reviews remained qualitatively unchanged from 2009 to 2013 (Table 5).

**Table 5:** Comparison of 2009 and 2013 content and methodological characteristics

	2009 Overall *N* = 793	2013 Overall *N* = 1293	Two proportion z-test *p*-value
Publication Characteristics			
Number of years between publication of protocol and review^a^ (median [IQR])	2 (1,3)	2 (1,3)	1.000
Nature of intervention^b^: classification 1, n (% total)			<0.001*
Pharmacological	468 (59.0)	666 (51.8)	0.001
Non-pharmacological	277 (34.9)	466 (36.2)	0.548
Both pharmacological and non-pharmacological	48 (6.1)	155 (12.0)	<0.001*
Nature of intervention^b^: classification 2, n (% total)			<0.001*
Drug	431 (52.2)	575 (44.5)	<0.001*
Vaccine	30 (3.6)	37 (2.9)	0.375
Natural Health Product	66 (8.0)	120 (9.3)	0.309
Surgical/clinical	76 (9.2)	53 (4.1)	<0.001*
Educational/behavioral/psychological/policy/legislative	140 (16.9)	169 (13.1)	0.017
Device	39 (4.7)	96 (7.4)	0.014
External source of funding^b^, n (% total)			0.010
Not stated	203 (25.6)	262 (20.3)	0.005
No	174 (21.9)	331 (25.6)	0.058
Yes	416 (52.5)	700 (54.1)	0.460
Government	281 (48.6)	461 (35.7)	<0.001*
Foundation/NPO	89 (15.4)	235 (18.2)	0.139
Cochrane	73 (12.6)	142 (11.0)	0.316
Study Designs			
Intended^b^			0.195
RCTs only (intended), n (% total)	430 (54.2)	695 (57.7)	0.118
RCTs and other designs (intended), n (% total)	360 (45.4)	502 (41.7)	0.098
Non-RCTs (intended), n (% total)	3 (0.4)	8 (0.7)	0.384
Actual^b^			0.017
RCTs only (actual), n (% total)	515 (71.6)	844 (73.5)	0.344
RCTs and other designs (actual), n (% total)	195 (27.1)	270 (23.5)	0.065
Non-RCTs (actual), n (% total)	9 (1.3)	34 (3.0)	0.013
Studies and participants			
Number of studies included^a^ (median, IQR)	7 (3, 15)	8 (4, 17)	0.001
Reviews with no relevant studies, n (% of reviews in group)	74 (9.3)	127 (9.8)	0.707
Number of participants included^a^ (median [IQR])	679 (179, 2833)	980 (278, 3394)	<0.001*
Outcomes			
Reviewers specified one or more primary outcomes, n (%)	574 (72.4)	1040 (82.3)	<0.001*
Analysis			
Children analyzed separately in reviews with both children and adult participants, n (%)	52 (11.5)	88 (10.0)	0.320
Subgroup analyses for children in reviews with both child and adult participants, n (%)	35 (5.3)	121 (13.9)	<0.001*
Publication bias assessed, n (%)	97 (12.2)	149 (13.8)	0.310
Meta-analysis conducted in those reviews with included studies, n (%)	483 (68.3)	784 (72.7)	0.040
Number of studies contributing to meta-analysis^a^ (median, IQR)	5 (3, 9)	5 (3, 10)	0.140

*Significant at Bonferroni corrected significance level *p* < 0.001

^a^Wilcoxon-Mann-Whitney test

^b^Chi-squared test

### General characteristics comparison

There was no difference in the number of years from protocol publication to review publication between 2009 and 2013 ([median 2; IQR: 1–3)] vs. [median 2; IQR: 1–3], respectively) (*p* = 1.000). The date for when reviews were ‘last assessed as up to date’ increased by a median of 3 years between 2009 to 2013 (2009: median date 2007; 2013: median date 2010). Between 2009 and 2013, the relative proportions of the nature of interventions studied in reviews (pharmacological interventions, non-pharmacological interventions, or both) changed in terms of clinical significance, but not statistical significance (*p* = 0.001). The largest change was in the proportion of reviews comparing pharmacological to non-pharmacological interventions (6.1% in 2009 vs. 12.0% in 2013) (*p* < 0.001). The proportion of reviews that had at least one source of funding was consistent between 2009 and 2013 (52.5% in 2009 vs. 54.1% in 2013) (*p* = 0.460).

### Characteristics of included studies comparison

The proportions of the types of study designs planned for inclusion in reviews (solely RCTs, RCTs and other designs, solely non-RCTs) remained constant from 2009 to 2013 (*p* = 0.195). There was no significant difference in the proportions of the types of study designs actually included in reviews from 2009 to 2013 (*p* = 0.017). Additionally, the median number of primary studies comprising each review did not increase significantly (2009: 7 [IQR: 3, 15] vs. 2013: 8 [IQR: 4, 17]) (*p* = 0.001). The median number of participants included per review, however, did increase significantly from 679 (IQR: 179, 2833) in 2009 to 980 (IQR: 278, 3394) in 2013 (*p* < 0.001).

### Methodological approaches comparison

The proportion of reviewers identifying a primary outcome increased by 10% from 2009 to 2013 (72.4% vs. 82.3%; *p* < 0.001). The proportion of reviews that analyzed children separately from adults was consistent between 2009 and 2013 (11.5% vs. 10.0%; *p* = 0.320). In mixed reviews, the proportion of reviews that conducted a subgroup analysis for children increased by 8.6% from 2009 to 2013 (*p* < 0.001). The proportion of reviews conducting a subgroup analysis within child data increased from 5.3% in 2009 to 25.6% in 2013 (*p* < 0.001). The proportion of reviews formally assessing publication bias did not increase significantly from 2009 to 2013 (12.2% vs. 13.8%, respectively; *p* = 0.310). The proportion of reviews that conducted at least one meta-analysis increased insignificantly by 4% from 2009 to 2013 (68.3% vs. 72.7%; *p* = 0.040). The median number of primary studies included in the largest meta-analysis remained unchanged from 2009 to 2013 (5 [IQR:3, 9] vs. 5 [IQR: 3, 10]) (*p* = 0.140).

### Review topics and global burden of disease

In 2013, 68% of the burden of disease in children aged 0–19 years could be attributed to the top 10 leading causes of death globally [13]. Among the 25 leading causes of death globally, the Review Groups associated with the most causes were: 1) ID, 2) Anaesthesia, Critical, and Emergency Care (ACE), 3) Injuries, 4) Pregnancy and Childbirth (PC), and 5) Neonatal (Additional file 3). There were large discrepancies among the number of causes of mortality that we associated with each Review Group and the proportion of evidence each Review Group contributed to the CHFRR. For example, while the ID Group was associated with 11 (44.0%) of the 25 leading causes of death, the ID Group contributed to only 79 (6.1%) of the reviews in the CHFRR. The greatest discrepancies were for the Review Groups: ID; ACE; Injuries; PC; Public Health; Wounds; and Drugs and Alcohol.

Given that it is desirable for disease burden and mortality rates to guide research prioritization, we might expect the Review Groups containing the largest number of reviews to also be the Review Groups most frequently associated with the top causes of mortality. Therefore, based on the Review Groups that were most frequently associated with each of the leading causes of mortality in 2013, we might expect the largest Review Groups to include: ARI (ranked in CHFRR: 3rd); Neonatal (not included); PC (ranked: 10th), ACE (ranked: 13th), ID (ranked: 5th); Inflammatory Bowel Diseases (IBD) (ranked: 14th); CF and Genetic Disorders (ranked: 2nd); Metabolic and Endocrine Disorders (ranked: 21st); Public Health (ranked: 39th); Injuries (ranked: 11th); Wounds (ranked: 18th); and Bone, Joint and Muscle Trauma (ranked: 27th) (Additional file 4).

The burden of childhood illness was overwhelmingly high in developing compared to developed countries. Ninety-eight percent of the cases of mortality in 2013 were from developing nations; however, only 224 (17.3%) reviews had corresponding authors from developing countries [13]. Corresponding authors from high-income countries were most often from the Review Groups: Airways, CF and Genetic Disorders, DPLP, ARI, and ID. Corresponding authors from low and middle-income countries were most often from the Review Groups: ID; ARI; HIV/AIDS; CF and Genetic Disorders; and DPLP.

## Discussion

Since 2009, the number of reviews in the CDSR has increased by 41% while the number of child-relevant reviews has increased by a modest 24%. This finding is consistent with evidence in the literature that the number of adult primary studies is increasing at a faster rate than the number of pediatric primary studies [18]. Less than a quarter (21.9%) of the primary studies included in child-relevant reviews restricted their participant inclusion criteria to children only. Among mixed reviews (1038 [77.6%]), only a minority (117 [11.3%]) of reviews analyzed children and adults separately and/or used subgroup analyses to differentiate between the effects in the two age groups (164 [15.8%]). This is particularly problematic given that it is widely accepted that children and adults differ not only in terms of their disease processes, physiology, and biology, but also in terms of their response to therapies [19]. Given that systematic reviews with both child and adult participants should be analyzing children separately or using subgroups [2], it is concerning that little change was observed in the proportion of reviews conducting separate or subgroup analyses for children between 2009 and 2013.

Several observations can be made with regards to the scope of the available primary research for child-relevant systematic reviews. Overall, 9.8% of systematic reviews were empty reviews. This represents a meaningful proportion of child-relevant reviews for which primary research is urgently needed in several topic areas. Of further concern, the median number of studies per review, which was 8 (IQR: 4, 17), was consistent with the findings of the 2009 CHFRR and Moher’s 2007 analysis of Cochrane reviews [2, 20]. This is often an insufficient amount of data to have confidence in the precision of results, to perform subgroup analyses, or to evaluate publication bias [2]. Finally, a median of 5 (IQR: 3, 10) studies made up the largest meta-analysis per review. This highlights that a large proportion of meta-analyses will only reflect a subset of the total available evidence in a review [2]. Further analysis of the reasons that studies do not contribute to the largest meta-analysis is needed in order to determine whether the studies are not assessing the same outcomes, not reporting results, or reporting results in a way that cannot be used for the purposes of meta-analysis. Inconsistent use of outcomes and reporting of outcome measures across primary studies is a recognized problem that becomes amplified at the systematic review level, and can impact the evidence available for decision-making [21–28]. The COMET initiative [29] aims to address these challenges by supporting efforts in the development and application of ‘core outcome sets’ (standardised outcome sets) within specific clinical areas.

A number of factors have been identified that may motivate authors to undertake an SR of a particular topic. Resolving conflicts between evidence, addressing questions of clinical uncertainty, exploring variations in practice, and highlighting the need for further research in a topic area have all been identified as potential motivating factors. Selecting and prioritizing research topics for SRs has become a challenging but essential task. Historically, Cochrane Review Groups have involved clinicians, researchers, and funding organizations in selecting research topics of priority [30].

In January 2015, the Cochrane Priority Reviews List project was launched. It is a ‘living’ record of Cochrane’s attempt to identify SR topics that are of greatest importance to stakeholders and that are most likely to impact health outcomes worldwide. A revised version of the list is published on a bi-monthly basis [31]. We found that the Review Groups that were most commonly associated with the leading causes of mortality in children in 2013 were often not the largest Review Groups in the CHFRR (i.e., Review Groups with the most reviews). Previous researchers studying the topic of SRs have also reported only a moderate correlation between the publication of review evidence and disease burden [12, 32, 33]. Further research is needed to evaluate whether the Cochrane Priority Reviews List has encouraged authors to produce reviews whose content more closely matches the global burden of disease in children.

The debate about the validity of including observational studies in systematic reviews that aim to estimate the effectiveness of interventions has existed for decades [34]. In general, RCTs are regarded as the ‘gold standard’ for the evaluation of prophylactic and therapeutic interventions [35]. The majority of child-relevant Cochrane systematic reviews continue to include RCTs only (73.5% in 2013 compared to 71.6% in 2009). Cochrane’s continued focus on RCTs reflects that Cochrane reviews aim to answer questions concerning the effectiveness of healthcare interventions [17, 36]. Other practical considerations are also a motivating reason for the restriction of numerous Cochrane SRs to RCTs [17]. There have been, however, criticisms regarding Cochrane’s focus on RCTs. In particular, whether RCTs are adequate for evaluating safety and real-world effectiveness has been a topic of much debate [34, 35, 37]. Another important point that has been raised by critics, especially in the context of child-relevant SRs, is that reviewers (and Review Groups) should consider expanding their inclusion criteria beyond RCTs to provide some or additional evidence for decision-making given that the number of studies included in a review is typically small and the proportion of reviews with no relevant studies (9.8%) is less than ideal [35, 37].

Diagnostic tests are a critical component of health care for diagnosing and establishing prognosis of disease. To ensure optimal patient care, practitioners must have an understanding of the true accuracy and efficacy of diagnostic tests, if testing improves outcomes, what tests to use, and how to interpret test results [38, 39]. SRs and meta-analyses of diagnostic studies, particularly pertaining to child health where studies are typically small, may enable the obtainment of more precise estimates of diagnostic accuracy. The first diagnostic test accuracy review was published in the CDSR in 2008. Although SRs of diagnostic test accuracy are increasingly being published, they continue to be methodologically difficult. Reviews of diagnostic test accuracy face two major challenges. The first challenge is the limited quality and availability of primary test accuracy studies. The second challenge is the need for further development in the areas of interpretation and presentation of the results of diagnostic test accuracy reviews [38, 39]. The CHFRR compiled in our study included a meagre total of 17 (1.3%) reviews of diagnostic accuracy. Given the large role diagnostic tests play in clinical decision-making, there continues to be a deficiency of Cochrane reviews examining diagnostic accuracy in child health.

A key goal of the Cochrane Collaboration is to make certain that all available evidence in the CDSR is up-to-date. To ensure this goal is reached, the Cochrane Collaboration has employed a policy that SRs contained in the CDSR be updated every two years [17]. A median date of 2010 was reported for when reviews in the CHFRR were “Last Assessed as Up-to-Date”, suggesting that the most reviews failed to meet the Cochrane policy at the time of this study. Over half (54%) of reviews were “Last Assessed as Up-to-Date” prior to 2011, and thus regarded as out of date according to Cochrane standards. This likely reflects the challenges in terms of time and resources required for updates that review authors and teams face, as well as other competing priorities. Previous research has focused on identifying signals for when updates are required [40–45]. An empirical approach to updating may be more appropriate to target scarce resources rather than a set policy of every two years for all reviews.

The proportion of reviews examining pharmacological interventions alone, non-pharmacological interventions alone, or both are consistent with the findings of the 2009 CHFRR and with Moher’s analysis of Cochrane SRs published in 2007 [2, 20]. The most often reviewed non-pharmacological interventions were behavioural, educational, psychological, or non-surgical clinical practices or procedures. This finding illustrates the usefulness of Cochrane reviews to many different audiences, such as, practitioners and policy-makers. An interesting finding of this study was the observation of a meaningful increase in the proportion of SRs comparing pharmacological to non-pharmacological interventions. This may reflect changes in the choices and decisions faced by healthcare decision-makers.

The Cochrane Collaboration is recognized for its inclusion of methodological experts worldwide and for its production of innovative systematic review methods [2]. The Cochrane Handbook for Systematic Reviews of Interventions represents the gold standard for systematic review methods [17]. It has been shown that Cochrane systematic reviews have more consistent and rigorous reporting quality than reviews published in other peer-reviewed journals [20, 36, 46–50]. Despite this, when the findings of the CHFRR were published in 2009, authors found that there was a large amount of inconsistency across Review Groups with regards to the preparation and conduct of reviews [2]. Although improvements have been made in some areas, the findings of the 2013 CHFRR demonstrate that there continues to be a substantial amount of variation in the preparation and conduct of SRs in the CHFRR. For example, while the proportion of reviewers not specifying a primary outcome decreased from 27.6% in 2009 to 17.7% in 2013, this continues to be unacceptably high.

Author’s approach to the assessment of methodological quality was also heterogeneous across reviews and Review Groups. The Cochrane Risk of Bias tool was the most commonly used (56.4%). This finding is consistent with our expectations given Cochrane’s endorsement of the use of the Cochrane Risk of Bias tool in 2008. Modified versions of the Cochrane Risk of Bias tool were sometimes used (7.7%). Allocation concealment was still widely used (24.6%), which is consistent with the out-dated methods recommended by Cochrane before 2008. It has been well established that scales should be used cautiously for the assessment of methodological quality [51]. In accordance with this recommendation, use of the Jadad scale decreased considerably since 2009 [2]. A small portion of reviews formally assessed publication bias, which was consistent with the previous findings of Bow et al. (2009) and Moher (2007) [2, 20]. Almost half of reviews did not make any attempt to discuss or assess publication bias. Approximately one third of reviews planned to assess publication bias, but did not carry out the assessment, which is likely due to the common problem of an inadequate number of studies included in a review for many child-relevant reviews.

Over half of reviews had one or more external sources of funding. The proportion of reviews having at least one external source of funding increased insignificantly by a small amount (1.6%) since 2009. This finding is important because funding is essential to ameliorating the substantial challenges of time and resources necessary to produce a methodologically rigorous systematic review. Ongoing funding is essential to the efforts of synthesizing evidence in child-health such that healthcare decisions can be established on the most rigorous and up-to-date research evidence.

The data reported here has built on the work conducted by Bow et al. [2]. Using the data compiled by Bow et al. as a baseline for comparison, we have examined changes over time in review methods, and the availability of child-relevant evidence in the CHFRR [2]. This work lays a foundation for which future researchers may build in regards to the identification of clinical topics areas that should be prioritized in the publication of systematic reviews.

### Limitations

The most challenging limitation encountered by the authors in this study was the inconsistency of reporting for key variables by review authors. This is similar to findings from previous studies which commented on the need to improve reporting of SRs involving children, and suggests that reporting quality continues to be a concern in Cochrane SRs [2, 49]. For example, a major limitation encountered in this study was the inconsistency of reporting for the total number of participants included in each SR. In many instances, there was unclear or no reporting of the total number of participants. Data extractors, in this situation, had to calculate the overall number of participants by hand from the information provided in the Characteristics of Included Studies Table. A further complication with tallying the number of participants was that review authors varied in their definitions of the number of participants included. Some authors reported the number corresponding to the number of participants recruited, while others reported the number corresponding to the number randomized, or analyzed. In cases where authors did not specify what the number of participants corresponded to, it was often challenging or impossible to distinguish. Therefore, the total number of participants reported in our study is likely an underestimate of the true number of participants included in all child-relevant SRs in the CHFRR.

In addition, the age range of participants of the included studies was reported inconsistently, or often not reported at all. This has important generalizability implications for end-users of systematic reviews. This is even more critical in the area of child health where children may respond differently to interventions across the age continuum (i.e., from 0 to 18 years). It is likely, though, that some of these reporting issues reflect limitations and variability in reporting of primary studies.

To facilitate the interpretation of changes over time, we chose to employ the same inclusion criteria of the former Cochrane Child Health Field Reviews Register. A limitation of using this inclusion criteria, however, is that it excludes pregnancy studies, with the exception of those assessing breastfeeding or nutritional supplements. Therefore, this study did not capture SRs pertaining to interventions administered during pregnancy and their effect on infant outcomes; this may be an interest for future work. In addition, reviews related to neonates were also not included in this descriptive analysis.

A further limitation of this study is that due to its cross-sectional design, the description and findings of this study provide a snapshot of child-relevant SRs in the CHFRR at a singular point in time. The content and methodological approaches of child-relevant Cochrane reviews are therefore likely to have changed between the time this sample was collected (CDSR Issue 3) and the time of publication of this study.

This study presents an overview of child-relevant SRs contained in the CDSR in 2013. Our study is not representative of all reviews relevant to child-health as SRs outside of the Cochrane Collaboration are increasingly being produced. A study produced in 2007 examining the epidemiology and reporting of SRs identified in Medline found that only one-fifth of the reviews in their sample were Cochrane reviews [20]. Since non-Cochrane reviews contribute to decision-making, future work in describing non-Cochrane child-relevant reviews would be valuable.

Finally, we provided additional analyses based on peer-reviewer feedback to examine the content of the CHFRR relative to global burden of disease and the extent of evidence relevant to high and middle/low income countries. These analyses were post-hoc. Further, we used proxy variables to examine these issues. We used the corresponding author’s country affiliation as a proxy for relevance of the topic to high vs. middle/low income countries, and number of reviews contained in different Review Groups as an indication of extent of evidence. The number of reviews does not necessarily reflect whether or not topics of most priority have been reviewed, which would require more in-depth analyses that were beyond the scope of this project.

## Conclusions

Less than a quarter of the primary studies included in Cochrane child-relevant reviews contained child-only populations; moreover, the average number of primary studies included per review in child-relevant systematic reviews and meta-analyses (8 and 5, respectively) is small and may limit decision-making for specific populations. There is a continued need for child-specific evidence, especially when it is suspected that efficacy and safety differ by age groups. Methods standardization should continue to be encouraged. The use of subgroups and separate analyses to present child data in reviews that contain mixed populations continues to be underutilized. Many reviews are out-of-date as per Cochrane updating recommendations; other strategies for signalling when updates are necessary should be explored to ensure the most efficient use of scarce resources.

There have also been advances in methods since 2009. These advancements include an increase in the number of review authors specifying a primary outcome and the number of author’s using the recommended Cochrane Risk of Bias tool to assess methodological quality. The CHFRR offers an important resource for understanding child-relevant systematic review research, and for clinical decision-making, by synthesizing a substantial extent of primary research. Further content analysis, combined with priority setting exercises, will lead to the identification of SR clinical topic areas of greatest priority for future SRs.

## Additional files

Additional file 1: Search Strategy, Database search strategy (DOC 22 kb)

Additional file 2: Screening Algorithm, Screening Algorithm for Inclusion of Reviews in the Child Health Field Review Register (DOC 102 kb)

Additional file 3: Table S6. Review Groups Identified as Applicable to the Top 25 Leading Causes of Mortality Globally in 2013 and the Proportion of Evidence in the CHFRR (DOCX 14 kb)

Additional file 4: Table S7. Ranking of Top 25 Leading Causes of Death in 2013 Globally, in Developing Nations, and Developed Nations (DOCX 19 kb)

## Abbreviations

ACE: Anaesthesia, Critical and Emergency Care
ARI: Acute Respiratory Infections
CDSR: Cochrane Database of Systematic Reviews
CF and Genetic Diseases: Cystic Fibrosis and Genetic Diseases
CHFRR: Child Health Field Review Register
CRGs: Cochrane Review Groups
DPLP: Developmental, Psychosocial and Learning Problems
GRADE: Grading of Recommendations Assessment, Development and Evaluation
H: High-income
IBD: Inflammatory Bowel Diseases
ID: Infectious Diseases
IQRs: Interquartile ranges
L/M: Low and middle-income
PC: Pregnancy and Childbirth
RCTs: Randomized controlled trials
RoB: Risk of bias
SDs: Standard deviations
SRs: Systematic reviews
