# Supplementary material for: Descriptive analysis of cochrane child-relevant systematic reviews: an update and comparison between 2009 and 2013
Source: BMC Pediatr. 2017 Jul 11;17:155. doi: 10.1186/s12887-017-0908-7 (PMC5504752; doi:10.1186/s12887-017-0908-7)

Additional file 2. Screening algorithm for inclusion of reviews in Child Health Field Review Register


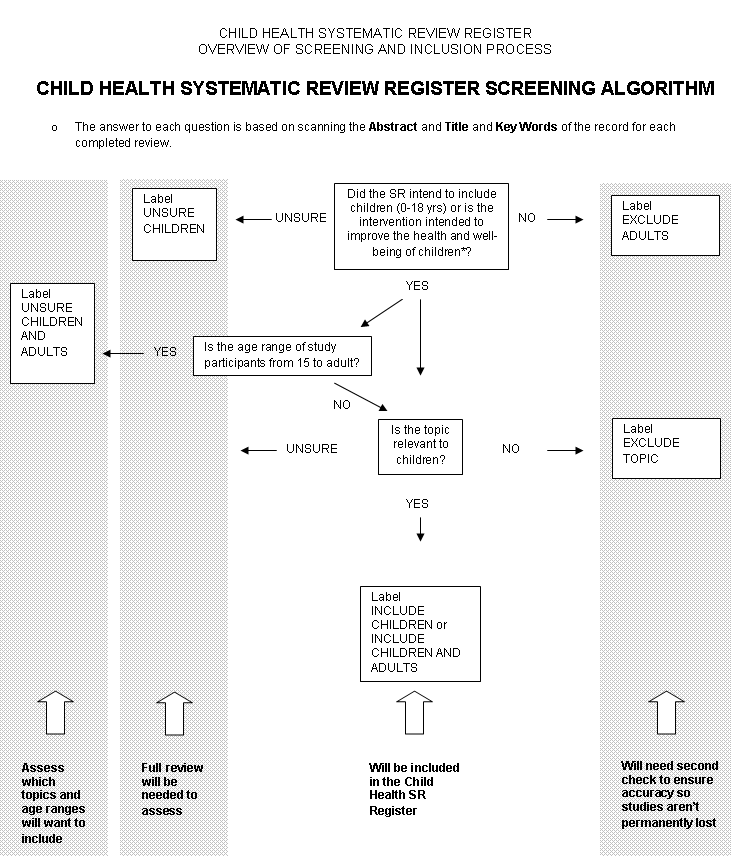


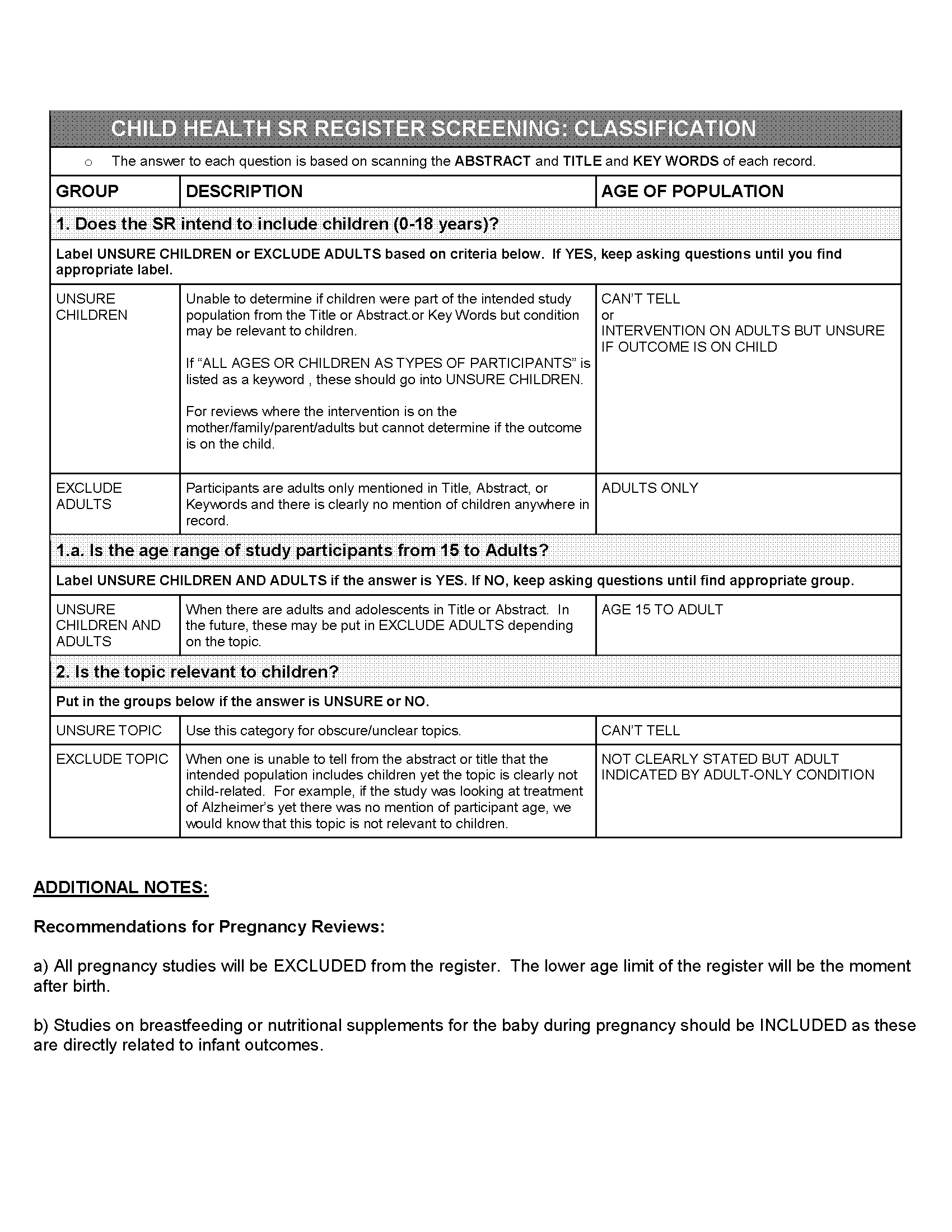

Supplement: Supplementary file 2 — Screening Algorithm, Screening Algorithm for Inclusion of Reviews in the Child Health Field Review Register (DOC 102 kb) [file 12887_2017_908_MOESM2_ESM.doc]
