# Supplementary material for: Descriptive analysis of cochrane child-relevant systematic reviews: an update and comparison between 2009 and 2013
Source: BMC Pediatr. 2017 Jul 11;17:155. doi: 10.1186/s12887-017-0908-7 (PMC5504752; doi:10.1186/s12887-017-0908-7)
Supplement: Supplementary file 3 — Review Groups Identified as Applicable to the Top 25 Leading Causes of Mortality Globally in 2013 and the Proportion of Evidence in the CHFRR (DOCX 14 kb) [file 12887_2017_908_MOESM3_ESM.docx]

**Additional file 3**

**Table S6.** Review Groups Identified as Applicalble to the Top 25 Leading Causes of Mortality Globally in 2013 and the Proportion of Evidence in the CHFRR

| **Review Groups** | **Number of Applicable Causes, n (%)** | **Proportion of evidence in the CHFRR, n (%)** |
| --- | --- | --- |
| Infectious Diseases | 11 (44.0) | 79 (6.1) |
| Anaesthesia, Critical, and Emergency Care | 7 (28.0) | 32 (2.5) |
| Injuries | 6 (24.0) | 38 (2.9) |
| Pregnancy and Childbirth | 5 (20.0) | 39 (3.0) |
| Neonatal | 4 (16.0) |  |
| Public Health | 4 (16.0) | 5 (0.4) |
| Wounds | 4 (16.0) | 27 (2.1) |
| CF and Genetic Disorders | 3 (12.0) | 103 (8.0) |
| Drugs and Alcohol | 3 (12.0) | 9 (0.7) |
| Acute Respiratory Infections | 2 (8.0) | 100 (7.7) |
| IBD | 2 (8.0) | 32 (2.5) |
| Metabolic and Endocrine Disorders | 2 (8.0) | 20 (1.6) |
| Developmental, Psychosocial, and Learning Problems | 2 (8.0) | 86 (6.7) |
| Depression, Anxiety, and Neurosis | 2 (8.0) | 21 (1.6) |
| Bone, Joint, and Muscle Trauma | 1 (4.0) | 16 (1.2) |
| HIV/AIDS | 1 (4.0) | 43 (3.3) |
| STI | 1 (4.0) | 0 (0.0) |
| Childhood Cancer | 1 (4.0) | 19 (1.5) |
| Skin | 1 (4.0) | 28 (2.2) |
