# Supplementary material for: Descriptive analysis of cochrane child-relevant systematic reviews: an update and comparison between 2009 and 2013
Source: BMC Pediatr. 2017 Jul 11;17:155. doi: 10.1186/s12887-017-0908-7 (PMC5504752; doi:10.1186/s12887-017-0908-7)
Supplement: Supplementary file 4 — Ranking of Top 25 Leading Causes of Death in 2013 Globally, in Developing Nations, and Developed Nations (DOCX 19 kb) [file 12887_2017_908_MOESM4_ESM.docx]

| **Additional file 4**  **Table S7.** Ranking of Top 25 Leading Causes of Death in 2013 Globally, in Developing Nations, and Developed Nations | |  | **Ranking** | |  |
| --- | --- | --- | --- | --- | --- |
| **Top 25 Global Causes of Death** | **Applicable Review Groups** | **Evidence in CHFRR, n (%)** | **Developing Countries** | **Developed Countries** | **Top 25 Largest Review Groups, n (%)** |
| 1. Lower Respiratory Tract Infections | ARI | 100 (7.7) | 1 | 7 | 1. Airways; 149 (11.5) |
| 1. Preterm Birth Complications | Neonatal, Pregnancy and Childbirth; Anaesthesia, Critical and Emergency Care | 71 (5.5) | 2 | 2 | 1. Cystic Fibrosis and Genetic Disorders; 103 (8.0) |
| 1. Malaria | Infectious Disease | 79 (6.1) | 3 | 130 | 1. Acute Respiratory Infections; 100 (7.7) |
| 1. Neonatal Encephalopathy | Neonatal, Pregnancy and Childbirth | 39 (3.0) | 4 | 6 | 1. Developmental, Psychosocial, and Learning Problems; 86 (6.7) |
| 1. Diarrheal Diseases | IBD; Infectious Diseases | 111 (8.6) | 5 | 26 | 1. Infectious Diseases; 79 (6.1) |
| 1. Congenital Anomalies | Pregnancy and Childbirth; CF and Genetic Disorders; Metabolic and Endocrine Disorders | 162 (12.5) | 6 | 1 | 1. Epilepsy; 50 (3.9) |
| 1. Neonatal Sepsis | Neonatal, Pregnancy and Childbirth; Infectious diseases | 118 (9.1%) | 7 | 13 | 1. Oral Health; 49 (3.8) |
| 1. Other Neonatal Disorders | Neonatal, Pregnancy and Childbirth | 39 (3.0) | 8 | 4 | 1. Ear, Nose, and Throat Disorders; 46 (3.6) |
| 1. Protein-Energy Malnutrition | Metabolic and Endocrine Disorders; Public Health, Infectious Disease; CF and Genetic Disorders | 207 (16.0) | 9 | 49 | 1. HIV/AIDs; 43 (3.3) |
| 1. Road Injuries | Injuries; Public Health; Wounds; Anaesthesia, Critical and Emergency Care; Bone, Joint, and Muscle Trauma | 118 (9.1) | 10 | 3 | 1. Pregnancy and childbirth; 39 (3.0) |
| 1. Meningitis | Infectious Disease | 79 (6.1) | 11 | 20 | 1. Injuries; 38 (2.9) |
| 1. HIV/AIDS | HIV/AIDS; Infectious Disease; Public Health | 127 (9.8) | 12 | 51 | 1. Renal; 38 (2.9) |
| 1. Hemoglobinopathies | Injuries; Drugs and Alcohol; Effective Practice and Organization of Care; Public Health; Anaesthesia, Critical, and Emergency Care | 97 (7.5%) | 14 | 9 | 1. Anaesthesia, Critical and Emergency Care; 32 (2.5) |
| 1. Drowning | CF and genetic disorders | 103 (8.0) | 13 | 36 | 1. IBD; 32 (2.5) |
| 1. Intestinal Infectious Diseases | Infectious Disease; IBD | 111 (8.6) | 15 | 34 | 1. Neuromuscular; 31 (2.4) |
| 1. Sexually Transmitted Diseases | Infectious Disease; STI | 79 (6.1) | 16 | 40 | 1. Pain, Palliation, and Supporting Care; 28 (2.2) |
| 1. Measles | Infectious Disease | 79 (6.1) | 17 | 102 | 1. Skin; 28 (2.2) |
| 1. Tuberculosis | Infectious Diseases | 79 (6.1) | 18 | 56 | 1. Wounds; 27 (2.1) |
| 1. Whooping Cough | ARI; Infectious Diseases | 179 (13.8%) | 19 | 67 | 1. Depression, Anxiety, and Neurosis; 21 (1.6) |
| 1. Mechanical Forces | Developmental, Psychosocial and Learning Problems; Drugs and Alcohol; Depression, Anxiety, and Neurosis; Anaesthesia, Critical, and Emergency Care | 148 (11.4) | 22 | 5 | 1. Eyes and Vision; 20 (1.6) |
| 1. Fire and Heat | Injuries; Wounds; Anaesthesia, Critical, and Emergency Care | 97 (7.5) | 20 | 17 | 1. Metabolic and Endocrine Disorders; 20 (1.6) |
| 1. Self-Harm | Injuries; Wounds; Anaesthesia, Critical, and Emergency Care | 97 (7.5) | 21 | 19 | 1. Tobacco Addiction; 20 (1.6) |
| 1. Foreign Body | Injuries; Wounds; Skin | 93 (7.2) | 23 | 16 | 1. Childhood Cancer; 19 (1.5) |
| 1. Interpersonal Violence | Injuries; Developmental, Psychosocial and Learning Problems; Depression, Anxiety, and Neurosis; Drugs and Alcohol; Anaesthesia, Critical, and Emergency Care | 186 (14.4) | 24 | 10 | 24. Consumers and Communication; 17 (1.3) |
| 1. Other Neoplasms | Childhood Cancer | 19 (1.5) | 25 | 11 | 25. Bone, Joint, and Muscle Trauma; 16 (1.2) |
